# A Comprehensive Genetic and Clinical Evaluation of Waardenburg Syndrome Type II in a Set of Iranian Patients

**DOI:** 10.22088/IJMCM.BUMS.7.1.17

**Published:** 2018-03-27

**Authors:** Nazanin Jalilian, Mohammad Amin Tabatabaiefar, Mahboubeh Yazdanpanah, Elham Darabi, Tayyeb Bahrami, Ali Zekri, Mohammad Reza Noori-Daloii

**Affiliations:** 1 *Department of Clinical biochemistry, School of Medicine, Kermanshah University of Medical Sciences, Kermanshah, Iran.*; 2 *Department of Genetics and Molecular Biology, School of Medicine, Isfahan University of Medical Sciences, Isfahan, Iran.*; 3 *Pediatric Inherited Diseases Research Center, Research Institute for Primordial Prevention of Noncommunicable Disease, Isfahan University of Medical Sciences, Isfahan, Iran.*; 4 *Department of Medical Genetics, School of Medicine, Tehran University of Medical Sciences, Tehran, Iran.*; 5 *Department of Medical Genetics, School of Medicine, International Campus, Tehran University of Medical Sciences, Tehran, Iran.*; 6 *Department of Medical Genetics and Molecular Biology, Faculty of Medicine, Iran University of Medical Sciences, Tehran, Iran.*

**Keywords:** Waardenburg syndrome type 2, Iran, MLPA, gene deletion, mutation

## Abstract

Waardenburg syndrome (WS) is a neurocristopathy with an autosomal dominant mode of inheritance, and considerable clinical and genetic heterogeneity. WS type II is the most common type of WS in many populations presenting with sensorineural hearing impairment, heterochromia iridis, hypoplastic blue eye, and pigmentary abnormalities of the hair and skin. To date, mutations of *MITF*, *SOX10*, and *SNAI2* have been implicated in the pathogenesis of WS2. Although different pathogenic mutations have been reported in many ethnic groups, the data on Iranian WS2 patients is insufficient. 31 WS2 patients, including 22 men and 9 women from 14 families were included. Waardenburg consortium guidelines were employed for WS2 diagnosis. WS2 patients underwent screening for *MITF*, *SOX10*, and *SNAI2* mutations using direct sequencing and MLPA analysis. Clinical evaluation revealed prominent phenotypic variability in Iranian WS2 patients. Sensorineural hearing impairment and heterochromia iridis were the most common features (67% and 45%, respectively), whereas anosmia was the least frequent phenotype. Molecular analysis revealed a *de novo* heterozygous c.640C>T (p.R214X) in *MITF* and a *de novo* heterozygous *SOX10* gross deletion in the study population. Our data help illuminate the phenotypic and genotypic spectrum of WS2 in an Iranian series of patients, and could have implications for the genetic counseling of WS in Iran.

Waardenburg syndrome (WS), coined by Dutch ophthalmologist Petrus Johannes Waardenburg, is a neurocristopathy composed of hearing impairment (HI) and pigmentary abnormalities of eyes, skin and hair (1). The syndrome is clinically and genetically heterogeneous, and follows an autosomal dominant mode of inheritance. Clinically, WS can be subdivided into four major forms WS1-WS4; distinguished by the presence/absence of additional features (2).

WS type II (WS2; OMIM:193510) is the most common type of WS in many populations (3, 4), presenting with sensorineural hearing impairment (SNHI), heterochromia iridis, hypoplastic blue eyes and pigmentary disorder of the hair. The clinical and genetic heterogeneity is evident in WS2; thus far, five subtypes of WS2 have been defined with distinct molecular etiologies. WS2A (MIM 193510; which is caused by mutations in *MITF*) (5), WS2B (MIM 600193; which maps on chromosome 1p) (6); WS2C (MIM 606662; which maps on chromosome 8p), WS2D (MIM 608890; caused by SNAI2 mutations) (7), and WS2E (MIM 611584; caused by SOX10 mutations*)* (8). The causative genes for WS2B and WS2C remain to be identified.

The mutations of aforementioned genes can only explain the etiology of 50% of WS2 cases (4). To date, several mutations have been identified related to WS in many populations. However, the Iranian population has been rarely investigated, with few reports being published on WS2. In fact, the only published report deals with a large WS2 kindred with unique spectrum of ocular findings and a novel *MITF* pathogenic variant (9). In this study, we performed a comprehensive clinical and genetic study on a set of Iranian WS2 cases, which led to better delineating phenotypic features, and revealed two pathogenic variants in *MITF* and *SOX10*.

## Materials and methods

**Participants**

In this case series study, patients were enrolled from the ENT and the Head and Neck Surgery Research Center, Iran University of Medical Sciences as well as several Special Education schools. Thorough clinical history was obtained and detailed audiological and ophthalmological examinations were carried out. All patients were diagnosed according to the Waardenburg Consortium criteria (10). The peripheral blood was collected after obtaining informed consent and DNA was extracted using a standard salting out method. This study was approved by the Ethics Committee of Tehran University of Medical Sciences.

**Mutation screening strategy**

WS2 patients were first analyzed for possible mutations of *SOX10*, *MITF*, and *SNAI2* using direct sequencing. Primer sequences to amplify all exons and exon-intron boundaries of *MITF*, *SOX10*, and *SNAI2* are available upon request. PCR products were subjected to bidirectional Sanger sequencing using an ABI3130 automated sequencer (Macrogen-South Korea). Sequence analysis was done using Chromas version2 (http://chromas. software.informer.com/2.0/).The sequence data were compared with RefSeqNM_000248, NM_006941.3 and NM_003068 for *MITF*, *SOX10*, and *SNAI2, *respectively. Upon finding a variant, databases including ensemble.org, dbSNP (http//:www.ncbi.hlm.nih.gov/snp), and 1000 genome databases (http//:browser.1000genome.org) were investigated. Samples negative for point mutation within candidate genes were then subjected to multiplex ligation probe amplification (MLPA) using P186-C2 PAX3 MITF SOX10 (MRC-Holland, Amsterdam, Netherlands). The MLPA mix contained probes to detect deletions/duplications in one or more sequences in the *PAX3*, *SOX10*, and *MITF* in a DNA sample. MLPA analysis was performed, as suggested by the manufacturer. Amplification products were run on an ABI PRISM 3130 Genetic Analyzer (Macrogen, South Korea) and the results were analyzed using the Gene Marker 2.0 Software. MLPA results were then confirmed using the quantitative real-time PCR. The sequences of the primers used for real-time analysis are provided in Table 1. Real-time PCR reaction was carried out on Rotor Gene 6000 Corbett instrument using 2X qPCR Master mix SYBR Green detection kits (RealQ plus master mix, amplicon, Denmark) with initial denaturation at 90 °C for 10 min, followed by 40 cycles of 95 °C for 15 s, 60 °C for 20 s, and 72 °C for 30 s. Fold changes in genomic copy number were calculated by ΔΔ Ct method. Hemoglobin subunit beta (HBB) was amplified as the reference gene. Melting curve analysis was applied to ensure specific reaction products.

## Results

**Clinical description**

Totally, 31 WS2 patients, including 22 men and 9 women from 14 families, and their healthy family members were enrolled. The study population comprised 5 familial and 9 sporadic cases ranging from 12 months to 77 years old.

**Fig. 1:**
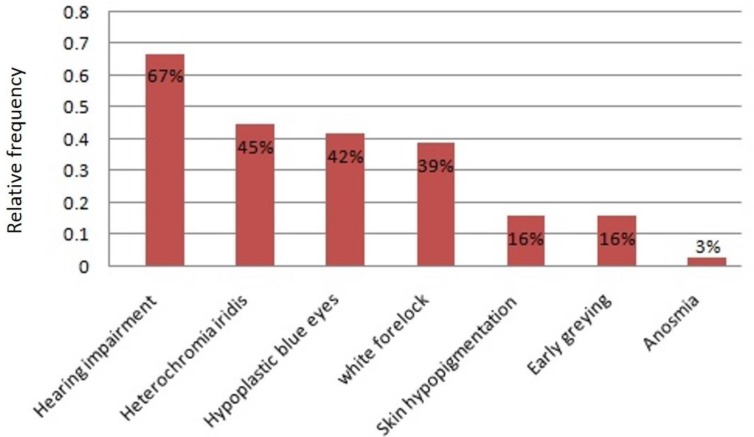
Distribution of WS2 associated phenotypes in the study population. Hearing impairment is the most frequent, and anosmia is the least frequent feature among Iranian WS2 patients.

HI was the most common clinical feature observed in our WS2 cases (67%, 21/31), followed by heterochromia iridis (45%, 14/31). HI was mostly observed as bilateral non-progressive severe to profound sensorineural; however, a case of WS2 with progressive SNHI was also observed. Distribution of WS2 clinical features among the WS2 study population is shown in Figure 1. As depicted in the figure, anosmia was the least frequent feature observed in only one patient.

**Table 1:** Oligonucleotide sequences of the primers used for real-time PCR

**Primer **	**Sequence 5' 3'**
F *SOX10*-Ex2	CTATCGGAGGTGGAGCTGAG
R *SOX10*-Ex2	GCTGCTCCTTCTTGACCTTGC
F *SOX10*-Ex3	CAGGCTGCTGAACGAAAGTGAC
R *SOX10*-Ex3	CAAGTGGGCGCTCTTGTAGTG
F *HBB*	GCTTCTGACACTACTGTGTT
R *HBB*	CACCAACTTCATCCACGTT

**Fig. 2:**
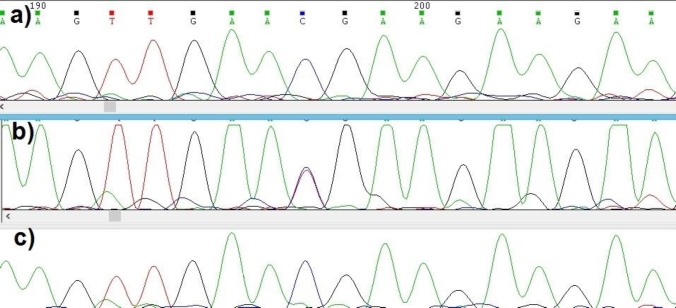
Electropherograms denoting the variant c.640C>T. a: normal father; b: proband; c: normal mother

**Fig. 3:**
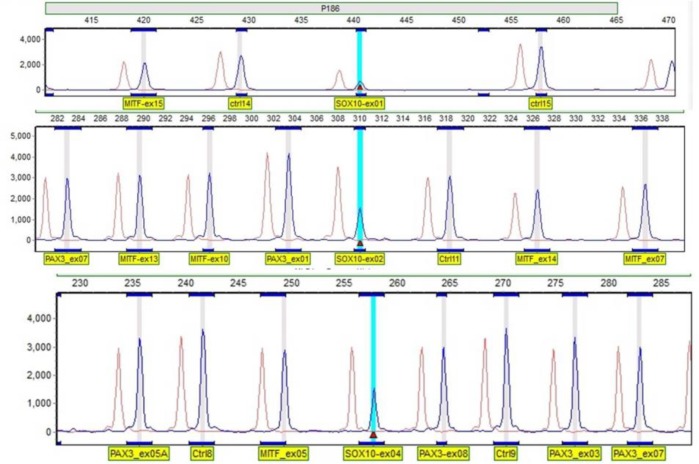
Results of MLPA analysis. MLPA analysis depicted whole SOX10 deletion in the proband of family IR-WS-08

**Fig. 4:**
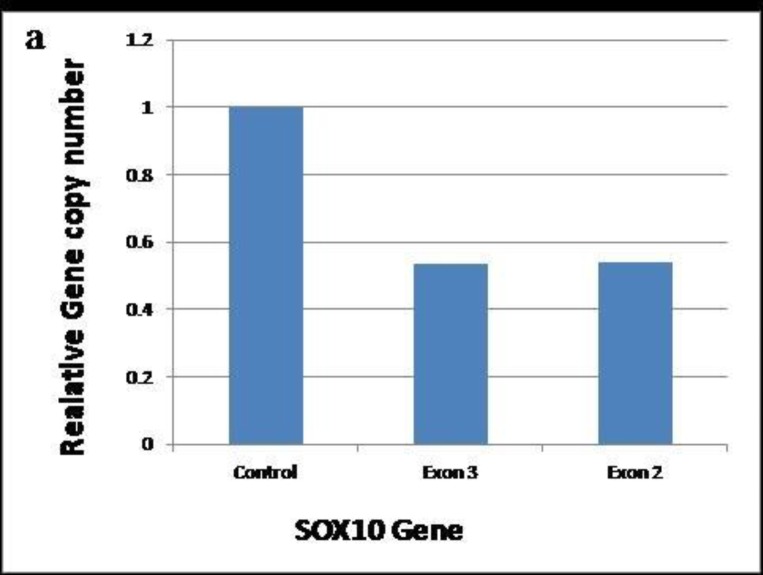
Relative copy number of SOX10 gene, determined by quantitative real-time PCR. Relative copy numbers represented as the fold change compared with a normal human. Values are shown as fold change in the relative copy number normalized with HBB on the basis of the 2- ΔΔ Ct method

**Spectrum of mutations**

Molecular analysis revealed a *de novo* heterozygous p.R214X mutation in *MITF*, and a* de novo* heterozygous *SOX10* gross deletion (exons 1-4) in the study population.

The *de novo* heterozygous variant c.640C>T (p.R214X at protein level) was identified in a 15 year old male with prelingual severe to profound sensorineural HI, corrected with cochlear implant, heterochromia iridis, and hypoplastic blue eyes with a W index<1.95; all suggestive of WS2 diagnosis. He was born to apparently normal, non-consanguineous parents. Their respective pedigree did not reveal any history of WS features in the 3 last generations.

Sequencing of all exons and exon-intron boundaries of *SOX10*, *MITF*, and *SNAI2* showed a *de novo* heterozygous variant c.640C>T in exon 7 of *MITF* in the proband while neither of the parents carried this variant (Figure 2). This nucleotide substitution introduces a stop codon at Arg214 in the protein basic domain, and has been reported previously in WS2A (11).

The proband 2, a 9 year old boy, was the second child of an unrelated healthy parent; her older sister was normal. At birth, he had hypotonia, and was admitted to hospital for severe seizure at age of 4.5 months. On physical examination, he presented with white forelock, white eyelashes, unilateral ptosis, and hypoplastic blue eyes while he had no history of hirschprung disease and hypo/hyperpigmented patches on the skin. He also showed abnormal tooth formation. Audiological examination revealed bilateral profound HI. Additionally, the proband exhibited several neurological symptoms, including ataxia and severe mental retardation. In order to detect any alteration of the myelination, further imaging investigation was advised which was refused by the parents.

The sequences of all exons and exon-intron boundaries of *SOX10*, *MITF*, and *SNAI2* were normal. However, the MLPA analysis showed a heterozygous deletion of all 4 exons of SOX10 in the proband (Figure 3), which was subsequently confirmed by real time PCR (Figure 4). No deletion/duplication was identified in the MITF and PAX3. This deletion was neither found in the three unaffected family members nor in the healthy controls.

## Discussion

WS2 which is inherited in an autosomal dominant manner, shares all WS1 phenotypes except for dystopia canthorum. WS2 shows considerable clinical heterogeneity. Studies suggest HI (77%), followed by heterochromia iridis (47%) as the most prevalent findings (12, 13); however, there might be slight differences among ethnic groups (13). Interestingly, the distribution of WS2 in our studied population followed the same pattern where HI and heterochromia iridis were the most prevalent findings among Iranian WS2 patients (frequencies of 67% and 45%, respectively).

There is only one *MITF* mutation described in Iranian WS2 cases so far (9), and here, we add another pathogenic variant to this list. The variant p.R214X, found in this study is located at basic domain of the protein, and abolishes the helix-loop-helix domain of the protein. Phenotype- genotype correlation has been speculated regarding non-truncating *MITF* basic domain mutations where Leger et al. reported a high frequency of ocular abnormalities (40%) among their studied patients with MITF basic domain mutations (14); however, it could not be applied to our study, since the truncating mutation was identified in one single patient.

*MITF* mutations are mostly private and among near 50 *MITF* mutations reported so far (http://www.hgmd.cf.ac.uk), few are known to be recurrent. Interestingly, we suggest p.R214X as a recurrent *MITF* variant since it has been reported three times since 1996 related to WS2. Our study presents the 4^th^ example (11, 15). On the other hand, due to the mutation which has been identified in both sporadic and familial cases, and that the familial cases did not share similar haplotypes, the site should be a mutational hot spot.

In this study, we failed to observe *SNAI2* mutations in the studied cases. Contribution of *SNAI2 *to the pathogenesis of WS2 was first described in 2002, where Sanchez-Martin et al. demonstrated two unrelated patients with homozygous *SNAI2* deletions (7). However, other studies could not confirm the involvement of *SNAI2* mutations in the pathogenesis of WS2 (16), suggesting a minor effect for this locus.

Deletions of the *SOX10 *has long been known to cause WS. Accordingly, here we demonstrated whole *SOX10* gene deletions in a WS2 patient susceptive to neurologic phenotype. Since the first report of *SOX10* haploinsufﬁency, due to gene deletions in WS (16), few patients with whole *SOX10* deletions have been presented. Siomou et al. reported a case with whole *SOX10* deletion who presented with the typical clinical features of WS2, and with a severe neurologic phenotype (17), while Wenzhi et al. showed* SOX10* whole gene deletion to be related to WS2 phenotypic spectrum with no neurological involvement (18). These lines of evidence reinforce the hypothesis that *SOX10* haploinsufﬁciency might be mainly related to HI and pigmentary abnormalities. Other molecular etiologies could account for the variability observed in the clinical and neurological presentations. Nevertheless, as few patients have been documented with whole *SOX10* deletions (http://www.hgmd.cf.ac.uk/ac/all.php), and little, molecular data is available on the deletions of other candidate genes within this region, patients with whole gene deletions would greatly help in figuring out function and contribution of these genes to neurologic phenotypes.

In conclusion, this study presents a comprehensive genetic and clinical investigation of WS2 in a group of Iranian patients. Nonetheless, the etiology was elucidated in 2 out of the 14 studied families. Further investigations of these cases using novel technologies (e.g. next-generation sequencing) could help greatly in unraveling the molecular mechanisms underlying WS in the Iranian population.
